# 2,2′-(Carbono­thio­yldisulfanedi­yl)bis­(2-methyl­propanoic acid)

**DOI:** 10.1107/S1600536813010179

**Published:** 2013-04-20

**Authors:** Rodolfo Moreno-Fuquen, Carlos Grande, Rigoberto C. Advincula, Juan C. Tenorio, Javier Ellena

**Affiliations:** a Departamento de Química, Facultad de Ciencias, Universidad del Valle, Apartado 25360, Santiago de Cali, Colombia; b Programa de Ingenieria Agroindustrial, Universidad San Buenaventura, AA 7154, Santiago de Cali, Colombia; c Case Western Reserve University, Department of Macromolecular Science and Engineering, 2100 Adelbert Road, Kent Hale Smith Bldg., Cleveland, Ohio 44106, USA; d Instituto de Física de São Carlos, IFSC, Universidade de São Paulo, USP, São Carlos, SP, Brazil

## Abstract

The mol­ecular structure of the title compound, C_9_H_14_O_4_S_3_, exhibits intra­molecular C—H⋯S hydrogen bonds. In the crystal, pairs of O—H⋯O hydrogen bonds lead to the formation of centrosymmetric dimers, which are in turn connected by weak C—H⋯O inter­actions. The combination of these inter­actions generates edge-fused *R*_2_^2^(8) and *R*_2_^2^(20) rings running along [211].

## Related literature

For pharmaceutical properties of tri­thio­carbonates, see: Dehmel *et al.* (2007 ▶). For tri­thio­carbonates as inter­mediates in organic synthesis, see: Metzner (1996 ▶). For the control of polymerization reactions of tri­thio­carbonates, see: Harrisson & Wooley (2005 ▶); Bilalis *et al.* (2006 ▶); Millard *et al.* (2006 ▶). For radical polymerization with RAFT reactions, see: Moad *et al.* (2005 ▶). For related structures, see: El-khateeb & Roller (2007 ▶). For hydrogen bonding, see: Nardelli (1995 ▶). For graph-set motifs, see: Etter (1990 ▶). For the synthesis, see: Lai *et al.* (2002 ▶).
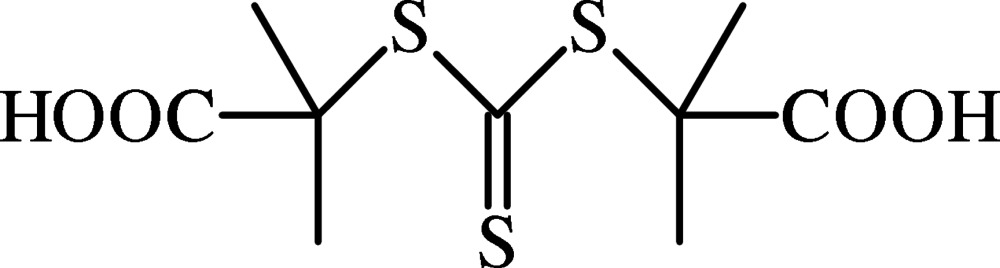


## Experimental

### 

#### Crystal data

C_9_H_14_O_4_S_3_
*M* *_r_* = 282.41
Monoclinic, 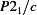

*a* = 10.4044 (2) Å
*b* = 10.4947 (2) Å
*c* = 13.7744 (3) Å
β = 117.363 (1)°
*V* = 1335.76 (5) Å^3^
*Z* = 4
Mo *K*α radiation
μ = 0.55 mm^−1^
*T* = 295 K
0.34 × 0.29 × 0.23 mm

#### Data collection

Nonius KappaCCD diffractometer
5423 measured reflections
2825 independent reflections
2273 reflections with *I* > 2σ(*I*)
*R*_int_ = 0.022

#### Refinement

*R*[*F* ^2^ > 2σ(*F* ^2^)] = 0.031
*wR*(*F* ^2^) = 0.084
*S* = 1.05
2825 reflections
151 parameters
1 restraint
H-atom parameters constrained
Δρ_max_ = 0.24 e Å^−3^
Δρ_min_ = −0.24 e Å^−3^

### 

Data collection: *COLLECT* (Nonius, 2000 ▶); cell refinement: *SCALEPACK* (Otwinowski & Minor, 1997 ▶); data reduction: *DENZO* (Otwinowski & Minor, 1997 ▶) and *SCALEPACK*; program(s) used to solve structure: *SHELXS97* (Sheldrick, 2008 ▶); program(s) used to refine structure: *SHELXL97* (Sheldrick, 2008 ▶); molecular graphics: *ORTEP-3 for Windows* (Farrugia, 2012 ▶) and *Mercury* (Macrae *et al.*, 2006 ▶); software used to prepare material for publication: *WinGX* (Farrugia, 2012 ▶).

## Supplementary Material

Crystal structure: contains datablock(s) I, global. DOI: 10.1107/S1600536813010179/zq2200sup1.cif

Structure factors: contains datablock(s) I. DOI: 10.1107/S1600536813010179/zq2200Isup2.hkl

Additional supplementary materials:  crystallographic information; 3D view; checkCIF report

## Figures and Tables

**Table 1:** Hydrogen-bond geometry (Å, °)

*D*—H⋯*A*	*D*—H	H⋯*A*	*D*⋯*A*	*D*—H⋯*A*
C8—H8*B*⋯S1	0.96	2.85	3.506 (2)	127
C5—H5*A*⋯S1	0.96	2.83	3.4955 (19)	127
O1—H1⋯O4^i^	0.82	1.84	2.6549 (17)	178
O3—H3⋯O2^i^	0.82	1.81	2.6321 (15)	178
C6—H6*C*⋯O4^ii^	0.96	2.69	3.518 (2)	144

Symmetry codes: (i) 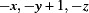; (ii) 
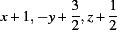
.

**Fig. 1.**
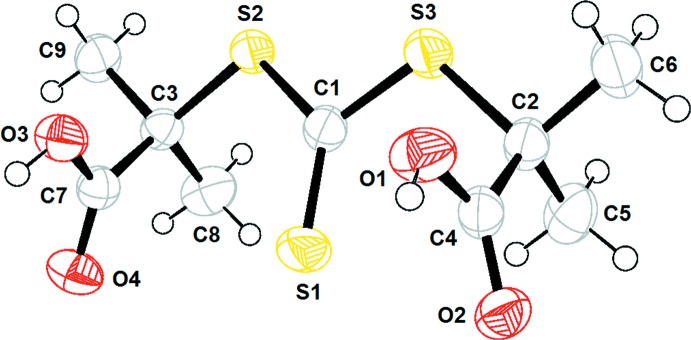
An ORTEP-3 (Farrugia, 2012) plot of the title compound with displacement ellipsoids drawn at the 50% probability level. H atoms are shown as spheres of arbitrary radius.

**Fig. 2.**
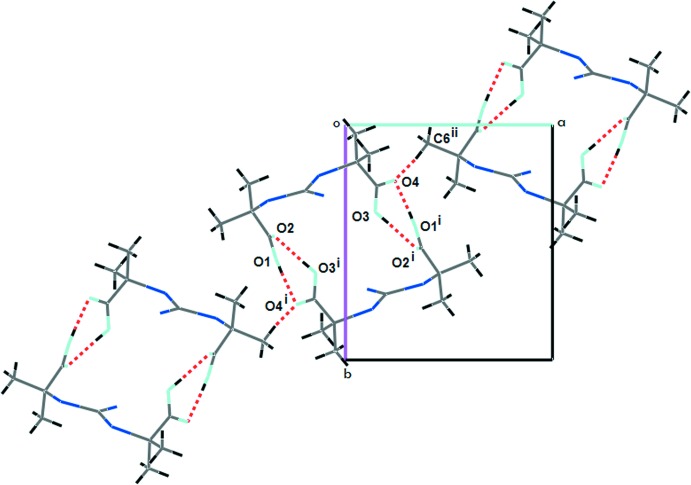
Part of the crystal structure of the title compound, showing the formation of chains of molecules running along [211]. Symmetry code: (i) -x,-y + 1,-z. (ii) x + 1,-y + 3/2,+z + 1/2.

## supplementary crystallographic information

### Comment

The title compound, C_9_H_14_O_4_S_3_, belongs to a series of organic trithiocarbonates that have received special attention due to their applications as pharmaceuticals (Dehmel *et al.*, 2007) or as intermediates in organic synthesis (Metzner *et al.*, 1996). Trithiocarbonates can be used to control the behavior of polymerization reactions (Harrisson & Wooley, 2005; Bilalis *et al.*, 2006; Millard *et al.*, 2006) or they are also used in radical polymerization with RAFT (reversible addition-fragmentation chain transfer) reactions (Moad *et al.*, 2005). A perspective view of the title compound (I), showing the atomic numbering scheme, is given in Fig. 1. The central trithio moiety in (I) is close to symmetric behavior. This behavior is different in an analogous structure (El-khateeb & Roller, 2007), where C1—S2 and C1—S3 bond lengths take values of 1.7733 (16) and 1.7232 (16) Å, respectively. This difference in the bond lengths is probably linked to the different ligand groups to which the trithio central group is connected. The title system shows intramolecular C—H···S interactions. The molecules of (I) are linked by O—H···O hydrogen bonds in their carboxyl terminals, forming centrosymmetric dimers. The O1 and O3 atoms at (*x*,*y*,*z*) act as hydrogen bond donors to O4 and O2 atoms of the carboxyl groups at (-*x*,-*y* + 1,-*z*). These dimers are connected to each other, through the weak C6—H6···O4, allowing them to grow along [211] (see Table 1, Nardelli, 1995). The C6 atom at (*x*,*y*,*z*) acts as hydrogen bond donor to O4 of the carboxy group at (*x* + 1,-*y* + 3/2,+*z* + 1/2). These intermolecular contacts are explained in terms of the substructure shown in Fig. 2. The combination of these interactions generate edge-fused *R*_2_^2^(8) and *R*_2_^2^(20) rings (Etter, 1990).

### Experimental

The compound (I) was synthesized according to a procedure reported in the literature (Lai *et al.*, 2002). Carbon disulfide (27.4 g, 0.361 mol), chloroform (107.5 g, 0.904 mol), acetone (52.3 g, 0.934 mol), and tetrabutylammonium hydrogen sulfate (2.41 g, 7.11 mmol) were mixed with 120 ml of mineral spirits in a 1 *L* round bottom flask under nitrogen. Sodium hydroxide (50%) was added dropwise over 90 min to maintain the temperature below 25°. The reaction was stirred overnight. 900 ml of water was added followed by 120 ml of concentrated HCl to acidify the aqueous layer. The mixture was filtered and rinsed with water. It was obtained a yellow cristalline solid which was purified with acetone. Mp. 447 (1) K. 2,2'-(thiocarbonylbis(sulfanediyl))bis(2-methylpropanoic acid), ^1^H-NMR (DMSO-d_6_, TMS): 1.58(s, 12H), 12.89(s, 2H). ^13^C-NMR(MeOD_4_): 25.74, 57.23, 176.23, 220.53. F T—IR (KBr): 3200–2800 (–COOH), 1711 (C=O), 1062 (–C=S), cm^-1^.

### Refinement

All H-atoms were placed in calculated positions [O—H = 0.82 Å and C—H = 0.96 Å for methyl group] and refined using a riding model approximation with *U*_iso_(H) constrained to 1.5 (O—H and methyl) times *U*_eq_ of the respective parent atom.

### Crystal data

**Table:**

C_9_H_14_O_4_S_3_	*F*(000) = 592
*M**_r_* = 282.41	*D*_x_ = 1.404 Mg m^−^^3^
Monoclinic, *P*2_1_/*c*	Melting point < 447(1) K
Hall symbol: -P 2ybc	Mo *K*α radiation, λ = 0.71073 Å
*a* = 10.4044 (2) Å	Cell parameters from 4448 reflections
*b* = 10.4947 (2) Å	θ = 2.9–26.4°
*c* = 13.7744 (3) Å	µ = 0.55 mm^−^^1^
β = 117.363 (1)°	*T* = 295 K
*V* = 1335.76 (5) Å^3^	Block, colourless
*Z* = 4	0.34 × 0.29 × 0.23 mm

### Data collection

**Table:**

Nonius KappaCCD diffractometer	2273 reflections with *I* > 2σ(*I*)
Radiation source: fine-focus sealed tube	*R*_int_ = 0.022
Graphite monochromator	θ_max_ = 26.7°, θ_min_ = 2.9°
CCD rotation images, thick slices scans	*h* = −13→13
5423 measured reflections	*k* = −12→13
2825 independent reflections	*l* = −17→17

### Refinement

**Table:**

Refinement on *F*^2^	Primary atom site location: structure-invariant direct methods
Least-squares matrix: full	Secondary atom site location: difference Fourier map
*R*[*F*^2^ > 2σ(*F*^2^)] = 0.031	Hydrogen site location: inferred from neighbouring sites
*wR*(*F*^2^) = 0.084	H-atom parameters constrained
*S* = 1.05	*w* = 1/[σ^2^(*F*_o_^2^) + (0.040*P*)^2^ + 0.2778*P*] where *P* = (*F*_o_^2^ + 2*F*_c_^2^)/3
2825 reflections	(Δ/σ)_max_ < 0.001
151 parameters	Δρ_max_ = 0.24 e Å^−^^3^
1 restraint	Δρ_min_ = −0.24 e Å^−^^3^

### Special details

**Table:**

Geometry. All e.s.d.'s (except the e.s.d. in the dihedral angle between two l.s. planes) are estimated using the full covariance matrix. The cell e.s.d.'s are taken into account individually in the estimation of e.s.d.'s in distances, angles and torsion angles; correlations between e.s.d.'s in cell parameters are only used when they are defined by crystal symmetry. An approximate (isotropic) treatment of cell e.s.d.'s is used for estimating e.s.d.'s involving l.s. planes.
Refinement. Refinement of *F*^2^ against ALL reflections. The weighted *R*-factor *wR* and goodness of fit *S* are based on *F*^2^, conventional *R*-factors *R* are based on *F*, with *F* set to zero for negative *F*^2^. The threshold expression of *F*^2^ > σ(*F*^2^) is used only for calculating *R*-factors(gt) *etc*. and is not relevant to the choice of reflections for refinement. *R*-factors based on *F*^2^ are statistically about twice as large as those based on *F*, and *R*- factors based on ALL data will be even larger.

### Fractional atomic coordinates and isotropic or equivalent isotropic displacement parameters (Å^2^)

**Table:**

	*x*	*y*	*z*	*U*_iso_*/*U*_eq_	
S1	0.10424 (5)	0.69853 (5)	0.04522 (4)	0.05515 (16)	
S2	0.12916 (4)	0.78396 (4)	0.26245 (3)	0.04274 (14)	
S3	0.37515 (4)	0.67719 (5)	0.26019 (3)	0.04675 (14)	
O1	0.33615 (14)	0.42986 (12)	0.13674 (9)	0.0503 (3)	
H1	0.3078	0.3696	0.0941	0.075*	
O2	0.34295 (12)	0.52637 (11)	−0.00505 (9)	0.0455 (3)	
O3	−0.14354 (12)	0.62947 (11)	0.14151 (9)	0.0445 (3)	
H3	−0.2066	0.5824	0.0979	0.067*	
O4	−0.23775 (13)	0.76463 (12)	0.00151 (9)	0.0483 (3)	
C1	0.19482 (17)	0.71974 (15)	0.17693 (13)	0.0381 (4)	
C2	0.45203 (17)	0.63284 (17)	0.16830 (13)	0.0419 (4)	
C3	−0.05423 (17)	0.84249 (15)	0.17297 (12)	0.0366 (3)	
C4	0.36796 (16)	0.52512 (16)	0.09110 (13)	0.0383 (4)	
C5	0.4659 (2)	0.74751 (19)	0.10631 (17)	0.0558 (5)	
H5A	0.3714	0.7811	0.0598	0.084*	
H5B	0.5115	0.7220	0.0627	0.084*	
H5C	0.5234	0.8118	0.1574	0.084*	
C6	0.60223 (18)	0.5785 (2)	0.24740 (15)	0.0561 (5)	
H6A	0.6495	0.5481	0.2063	0.084*	
H6B	0.5906	0.5095	0.2884	0.084*	
H6C	0.6598	0.6443	0.2966	0.084*	
C7	−0.15123 (16)	0.74014 (15)	0.09617 (13)	0.0361 (3)	
C8	−0.0491 (2)	0.96101 (17)	0.11115 (16)	0.0554 (5)	
H8A	−0.1453	0.9939	0.0695	0.083*	
H8B	−0.0093	0.9394	0.0627	0.083*	
H8C	0.0105	1.0244	0.1622	0.083*	
C9	−0.11435 (18)	0.87531 (18)	0.25362 (14)	0.0477 (4)	
H9A	−0.1148	0.8001	0.2931	0.072*	
H9B	−0.2113	0.9072	0.2138	0.072*	
H9C	−0.0544	0.9390	0.3041	0.072*	

### Atomic displacement parameters (Å^2^)

**Table:**

	*U*^11^	*U*^22^	*U*^33^	*U*^12^	*U*^13^	*U*^23^
S1	0.0460 (3)	0.0823 (4)	0.0357 (2)	0.0060 (2)	0.0175 (2)	−0.0126 (2)
S2	0.0379 (2)	0.0577 (3)	0.0339 (2)	−0.00402 (18)	0.01772 (18)	−0.01094 (18)
S3	0.0382 (2)	0.0629 (3)	0.0380 (2)	−0.00095 (19)	0.01655 (19)	−0.0126 (2)
O1	0.0637 (8)	0.0455 (7)	0.0407 (6)	−0.0137 (6)	0.0231 (6)	−0.0044 (5)
O2	0.0509 (7)	0.0515 (7)	0.0396 (6)	−0.0110 (6)	0.0254 (5)	−0.0085 (5)
O3	0.0501 (7)	0.0375 (7)	0.0386 (6)	−0.0082 (5)	0.0140 (5)	0.0010 (5)
O4	0.0501 (7)	0.0431 (7)	0.0375 (7)	0.0021 (5)	0.0079 (6)	0.0026 (5)
C1	0.0384 (8)	0.0397 (9)	0.0386 (7)	−0.0071 (7)	0.0197 (7)	−0.0083 (7)
C2	0.0368 (8)	0.0488 (10)	0.0438 (9)	−0.0072 (7)	0.0217 (7)	−0.0121 (8)
C3	0.0413 (8)	0.0358 (9)	0.0351 (8)	−0.0033 (7)	0.0195 (7)	−0.0046 (6)
C4	0.0327 (8)	0.0432 (9)	0.0411 (9)	−0.0018 (7)	0.0186 (7)	−0.0047 (7)
C5	0.0592 (12)	0.0519 (11)	0.0653 (12)	−0.0185 (9)	0.0363 (10)	−0.0126 (9)
C6	0.0375 (9)	0.0708 (13)	0.0572 (11)	−0.0017 (9)	0.0192 (8)	−0.0141 (10)
C7	0.0369 (8)	0.0361 (9)	0.0369 (9)	0.0004 (7)	0.0183 (7)	−0.0014 (7)
C8	0.0770 (13)	0.0376 (10)	0.0559 (11)	−0.0077 (9)	0.0342 (10)	−0.0017 (8)
C9	0.0470 (10)	0.0541 (11)	0.0478 (10)	−0.0012 (8)	0.0268 (8)	−0.0115 (8)

### Geometric parameters (Å, º)

**Table:**

S1—C1	1.6301 (16)	C3—C8	1.522 (2)
S2—C1	1.7460 (16)	C3—C9	1.544 (2)
S2—C3	1.8372 (16)	C5—H5A	0.9600
S3—C1	1.7484 (17)	C5—H5B	0.9600
S3—C2	1.8410 (16)	C5—H5C	0.9600
O1—C4	1.302 (2)	C6—H6A	0.9600
O1—H1	0.8200	C6—H6B	0.9600
O2—C4	1.2268 (18)	C6—H6C	0.9600
O3—C7	1.3037 (19)	C8—H8A	0.9600
O3—H3	0.8200	C8—H8B	0.9600
O4—C7	1.2238 (18)	C8—H8C	0.9600
C2—C5	1.520 (3)	C9—H9A	0.9600
C2—C4	1.523 (2)	C9—H9B	0.9600
C2—C6	1.546 (2)	C9—H9C	0.9600
C3—C7	1.520 (2)		
			
C1—S2—C3	106.51 (7)	C2—C5—H5C	109.5
C1—S3—C2	106.73 (7)	H5A—C5—H5C	109.5
C4—O1—H1	109.5	H5B—C5—H5C	109.5
C7—O3—H3	109.5	C2—C6—H6A	109.5
S1—C1—S2	126.75 (10)	C2—C6—H6B	109.5
S1—C1—S3	126.32 (9)	H6A—C6—H6B	109.5
S2—C1—S3	106.90 (9)	C2—C6—H6C	109.5
C5—C2—C4	111.58 (14)	H6A—C6—H6C	109.5
C5—C2—C6	111.21 (15)	H6B—C6—H6C	109.5
C4—C2—C6	106.77 (14)	O4—C7—O3	123.42 (15)
C5—C2—S3	111.67 (12)	O4—C7—C3	121.41 (15)
C4—C2—S3	112.10 (11)	O3—C7—C3	114.91 (13)
C6—C2—S3	103.10 (11)	C3—C8—H8A	109.5
C7—C3—C8	111.77 (14)	C3—C8—H8B	109.5
C7—C3—C9	107.43 (13)	H8A—C8—H8B	109.5
C8—C3—C9	110.50 (14)	C3—C8—H8C	109.5
C7—C3—S2	112.54 (11)	H8A—C8—H8C	109.5
C8—C3—S2	110.85 (12)	H8B—C8—H8C	109.5
C9—C3—S2	103.37 (11)	C3—C9—H9A	109.5
O2—C4—O1	123.69 (15)	C3—C9—H9B	109.5
O2—C4—C2	120.97 (15)	H9A—C9—H9B	109.5
O1—C4—C2	115.13 (13)	C3—C9—H9C	109.5
C2—C5—H5A	109.5	H9A—C9—H9C	109.5
C2—C5—H5B	109.5	H9B—C9—H9C	109.5
H5A—C5—H5B	109.5		
			
S1—S1—C1—S2	0.00 (2)	C1—S2—C3—C9	170.36 (11)
S1—S1—C1—S3	0.00 (5)	C5—C2—C4—O2	13.2 (2)
C3—S2—C1—S1	−7.38 (14)	C6—C2—C4—O2	−108.50 (17)
C3—S2—C1—S1	−7.38 (14)	S3—C2—C4—O2	139.30 (13)
C3—S2—C1—S3	174.63 (8)	C5—C2—C4—O1	−171.90 (14)
C2—S3—C1—S1	10.52 (14)	C6—C2—C4—O1	66.40 (17)
C2—S3—C1—S1	10.52 (14)	S3—C2—C4—O1	−45.80 (17)
C2—S3—C1—S2	−171.47 (8)	C8—C3—C7—O4	−15.6 (2)
C1—S3—C2—C5	68.46 (14)	C9—C3—C7—O4	105.74 (17)
C1—S3—C2—C4	−57.59 (14)	S2—C3—C7—O4	−141.14 (13)
C1—S3—C2—C6	−172.07 (12)	C8—C3—C7—O3	170.06 (14)
C1—S2—C3—C7	54.77 (13)	C9—C3—C7—O3	−68.56 (18)
C1—S2—C3—C8	−71.23 (13)	S2—C3—C7—O3	44.56 (16)

### Hydrogen-bond geometry (Å, º)

**Table:**

*D*—H···*A*	*D*—H	H···*A*	*D*···*A*	*D*—H···*A*
C8—H8*B*···S1	0.96	2.85	3.506 (2)	127
C5—H5*A*···S1	0.96	2.83	3.4955 (19)	127
O1—H1···O4^i^	0.82	1.84	2.6549 (17)	178
O3—H3···O2^i^	0.82	1.81	2.6321 (15)	178
C6—H6*C*···O4^ii^	0.96	2.69	3.518 (2)	144

Symmetry codes: (i) −*x*, −*y*+1, −*z*; (ii) *x*+1, −*y*+3/2, *z*+1/2.

## References

[bb1] Bilalis, P., Pitsikalis, M. & Hadjichristidis, N. (2006). *J. Polym. Sci. Part A Polym. Chem.* **44**, 659–665.

[bb2] Dehmel, F., Ciossek, T., Maier, T., Weinbrenner, S., Schmidt, B., Zoche, M. & Beckers, T. (2007). *Bioorg. Med. Chem. Lett.* **17**, 4746–4752. 10.1016/j.bmcl.2007.06.063 17606370

[bb3] El-khateeb, M. & Roller, A. (2007). *Polyhedron*, **26**, 3920–3924.

[bb4] Etter, M. C. (1990). *Acc. Chem. Res.* **23**, 120–126.

[bb5] Farrugia, L. J. (2012). *J. Appl. Cryst.* **45**, 849–854.

[bb6] Harrisson, S. & Wooley, K. L. (2005). *Chem. Commun.* pp. 3259–3261. 10.1039/b504313a 15983640

[bb7] Lai, J. T., Filla, D. & Shea, R. (2002). *Macromolecules*, **35**, 6754–6756.

[bb8] Macrae, C. F., Edgington, P. R., McCabe, P., Pidcock, E., Shields, G. P., Taylor, R., Towler, M. & van de Streek, J. (2006). *J. Appl. Cryst.* **39**, 453–457.

[bb9] Metzner, P. (1996). *Pure & Appl. Chem.* **68**, 863–868.

[bb10] Millard, P. E., Barner, L., Stenzel, M. H., Davis, T. P., Barner-Kowollik, C. & Muller, A. H. E. (2006). *Macromol. Rapid Commun.* **27**, 821–828.

[bb11] Moad, G., Chong, Y. K., Postma, A., Rizzardo, E. & Thang, S. H. (2005). *Polymer*, **46**, 8458–8468.

[bb12] Nardelli, M. (1995). *J. Appl. Cryst.* **28**, 659.

[bb13] Nonius (2000). *COLLECT* Nonius BV, Delft, The Netherlands.

[bb14] Otwinowski, Z. & Minor, W. (1997). *Methods in Enzymology*, Vol. 276, *Macromolecular Crystallography*, Part A, edited by C. W. Carter Jr & R. M. Sweet, pp. 307–326. New York: Academic Press.

[bb15] Sheldrick, G. M. (2008). *Acta Cryst.* A**64**, 112–122. 10.1107/S0108767307043930 18156677

